# Symptom Dimension Breakpoints for the Obsessive-Compulsive Inventory-Child Version (OCI-CV)

**DOI:** 10.1007/s10578-021-01305-4

**Published:** 2022-01-03

**Authors:** Matti Cervin, Blanca Garcia-Delgar, Rosa Calvo, Ana E. Ortiz, Luisa Lazaro

**Affiliations:** 1 Department of Clinical Sciences Lund, Child and Adolescent Psychiatry, Faculty of Medicine, Lund University, Sofiavägen 2D, SE-22241 Lund, Sweden; 2 Department of Child and Adolescent Psychiatry and Psychology, Hospital Clínic Universitari, Barcelona, Spain; 3 Department of Medicine, IDIBAPS, CIBERSAM, University of Barcelona, Barcelona, Spain

**Keywords:** OCD, Children, Adolescents, Symptom dimensions

## Abstract

Pediatric obsessive-compulsive disorder (OCD) clusters around three major symptom dimensions: contamination/cleaning, symmetry/ordering, and disturbing thoughts/checking. The Obsessive-Compulsive Inventory-Child Version (OCI-CV) is a self-report questionnaire that provides scores along six theory-based OCD dimensions, but no study has evaluated how well OCI-CV identifies clinically significant symptoms within each of the three major symptom dimensions of OCD. We examined this question using data from 197 Swedish and Spanish youth with OCD. All youth completed the OCI-CV and clinically significant symptom severity within each major OCD dimension was established with a validated interview-based measure. Results showed that a score ≥ 3 on the OCI-CV washing scale excellently captured those with clinically significant contamination/cleaning symptoms (AUC = 0.85 [0.80–0.90], 79% accuracy). A score ≥ 4 on the obsessing scale adequately captured those with disturbing thoughts/checking symptoms (AUC = 0.71 [0.64–0.78], 67% accuracy) and a score ≥ 3 on the ordering scale adequately captured those with symmetry/ordering symptoms (AUC = 0.72 [0.65–0.79], 70% accuracy). Similar accuracy of the breakpoints was found in the Swedish and Spanish samples. OCI-CV works well to identify youth with pediatric OCD that have clinically significant contamination/cleaning symptoms. The measure can also with adequate precision identify those with clinically significant disturbing thoughts/checking and symmetry/ordering symptoms. The breakpoints provided in this study can be used to examine differences in clinical presentation and treatment outcome for youth with different types of OCD.

## Introduction

Pediatric obsessive-compulsive disorder (OCD) affects 1–2% of children and adolescents and can run a chronic course without adequate treatment [1, 2]. Symptom onset of OCD occurs for most sufferers before adulthood [3] and research on pediatric OCD is important to better understand onset and clinical course. OCD is extremely heterogeneous, but factor analytic work has shown that OCD symptoms cluster around three broad symptom dimensions: contamination/cleaning, symmetry/ordering, and disturbing thoughts/checking [4]. A fourth dimension that includes obsessions and compulsions related to hoarding has also been identified, which partly led to hoarding symptoms being conceptualized as a distinct diagnostic category in DSM-5 [5]. A recent study, analyzing a more comprehensive pool of OCD symptoms, showed that there may be up to eight valid symptom dimensions of OCD [6].

In youth, the three major symptom dimensions of OCD are driven by partially distinct underlying emotions, that is, fear drives checking symptoms, disgust drives washing symptoms, and incompleteness drives symmetry-related symptoms [7, 8]. Moreover, cognitive beliefs proposed as important for the onset and maintenance of OCD (e.g. inflated sense of responsibility, dysfunctional metacognitions) are predominantly linked to harm/responsibility obsessions and checking compulsions and not to contamination and symmetry-related symptoms [9]. Heritability and neural correlates of symptoms have also been shown to differ across OCD symptom dimensions, but this evidence is almost exclusively based on research with adults [10–13]. Taken together, emerging evidence suggests that it may be crucial to account for symptom dimensions of OCD when trying to elucidate the onset, course, and causes of the disorder. As no biological tests exist that can help adequately assess symptom dimensionality of OCD, valid psychometric measurement is key.

Few measures of symptom dimensions of pediatric OCD exist [14]. In the self-report category, one exception is the Obsessive-Compulsive Inventory-Child Version (OCI-CV) [15]. OCI-CV is a 21-item scale that assesses symptoms within six theory-based symptom dimensions: doubting/checking, obsessing, washing, hoarding, ordering, and neutralizing. OCI-CV also provides an overall score that can be used to indicate overall severity of OCD. However, as this overall score pools items across symptom dimensions, it may be inadequate to capture overall symptom severity, particularly for patients with symptoms revolving around a small number of symptoms (e.g., only contamination symptoms). Limitations of the overall OCI-CV scale as a measure of broad symptom severity is supported by empirical research that shows that it correlates weakly with interview-based measures of symptom severity, such as the Children’s Yale-Brown Obsessive Compulsive Scale (CY-BOCS) [16]. The OCI-CV factor structure has shown good to excellent model/data fit across a range of studies with clinical and non-clinical children and adolescents from different countries, and the OCI-CV subscales, except for the neutralization scale, have shown good internal consistency [16–20]. Further, the total score of OCI-CV discriminates youth with OCD from those with anxiety and tic disorders [16]. A recent study, using a large sample of children and adolescents with and without psychiatric disorders, conducted semi-structured diagnostic interviews with all participants and showed that an OCI-CV total score equal to or above 11 showed promise to identify clinical OCD in psychiatric settings, while a score of 10 may be best in primacy care, where pediatric OCD is rarer [21].

OCI-CV is commonly used in research about pediatric OCD, including in clinical trials, but despite similarities, the OCI-CV scales were not developed to capture the three major symptom dimensions identified through factor analytic work of the child and adult versions of the symptom checklist of the Yale-Brown Obsessive Compulsive Scale (Y-BOCS) [22]. To evaluate how well OCI-CV captures severity within each of the major symptom dimensions of OCD, a reference measure is needed where symptom dimension severity is adequately assessed. The most comprehensive measure of OCD symptom dimension severity is the Dimensional Yale-Brown Obsessive-Compulsive Scale (DY-BOCS). It is a clinician-administered interview that assesses time, interference, and distress across six OCD symptom dimensions: contamination/cleaning, symmetry/ordering, disturbing thoughts/checking, sexual/religious obsessions/compulsions, hoarding, and a miscellaneous dimension [23]. The aim of this study is to evaluate how the OCI-CV subscales capture clinically significant symptoms within each of the three major symptom dimensions of OCD measured via DY-BOCS.

## Methods

### Participants, Procedure, and Ethics

We pooled two samples of children and adolescents that met diagnostic criteria for OCD. The first sample was collected as part of a Swedish study on cognitive and emotional mechanisms in pediatric OCD. All patients were seen at a specialized child and adolescent psychiatric unit in Southern Sweden. Diagnostic status was assessed via the structured diagnostic interview Mini International Neuropsychiatric Interview for Children and Adolescents [24]. All participants were invited to the study at intake, at which OCI-CV was completed and the DY-BOCS interview was used to assess symptom dimension severity. OCI-CV ratings were not known to the DY-BOCS interviewer. Data collection was conducted between 2015 and 2019. The second sample was collected as part of a Spanish genetics and neuroimaging study on pediatric OCD. Diagnostic status was assessed via the semi-structured diagnostic interview Kiddie Schedule for Affective Disorders and Schizophrenia [25]. Participants in this sample were at different stages of treatment and data collection was conducted between 2011 and 2014. Both studies were approved by ethical review boards in each country. Sociodemographic and clinical information is presented in Table 1.

**Table 1:** Sociodemographic and clinical information for the Swedish and Spanish sample

	Spain	Sweden	Combined
*n*	96	101	197
% Girls	50%	61%	56%
Age, *M* (*SD*)	14.8 (2.54)	13.4 (2.6)	14.1 (2.7)
Age, Min-max	8.2–18.9	8.0–17.8	8.0–18.9
CY-BOCS, *M* (*SD*)	18.4 (7.9)	23.3 (4.2)	20.9 (6.8)
CY-BOCS, Min-max	0–34	11–33	0–34
Clinically significant disturbing thoughts/checking symptoms	44%	62%	53%
Clinically significant symmetry/ordering symptoms	53%	56%	55%
Clinically significant contamination/cleaning symptoms	45%	53%	49%
OCI-CV Doubting/checking, *M* (*SD*)	3.96 (2.65)	4.96 (2.78)	4.47 (2.76)
OCI-CV Obsessing, *M* (*SD*)	3.81 (2.42)	4.49 (2.06)	4.16 (2.26)
OCI-CV Washing, *M* (*SD*)	2.13 (1.91)	2.87 (2.17)	1.81 (1.65)
OCI-CV Hoarding, *M* (*SD*)	1.94 (1.70)	1.70 (1.59)	2.51 (2.08)
OCI-CV Ordering, *M* (*SD*)	2.43 (2.15)	3.06 (2.11)	2.75 (2.15)
OCI-CV Neutralization, *M* (*SD*)	1.17 (1.36)	1.78 (1.61)	1.48 (1.52)

*CY-BOCS* Children’s Yale-Brown Obsessive Compulsive Scale, *OCI-CV* Obsessive Compulsive Inventory-Child Version

### Measures

#### OCI-CV

 OCI-CV is a self-report measure where children and adolescents report on the presence of 21 OCD symptoms on a 0–2 scale. Higher scores indicate more frequent symptoms with a 0 indicating *Never* and a 2 indicating *Always*. The measure consists of six subscales described in the introduction. Previous research has shown that OCI-CV has an adequate factor structure and acceptable internal consistency on the subscale level across different countries [15–18, 20]. Which subscale scores that best correspond to clinically significant symptom severity across the major symptom dimensions of pediatric OCD has never been examined. The Swedish and Spanish versions of OCI-CV used in this study have been evaluated in prior research [16, 17], indicating preserved psychometric properties compared to the original version [15].

#### DY-BOCS

The DY-BOCS is used to assess symptoms within the six OCD symptom dimensions outlined in the introduction. An interviewer carefully assesses the presence/absence of different OCD symptoms and then rate the severity of symptoms within each dimension using three questions about time, distress, and interference, each scored on a 0-5 scale with higher scores indicating higher severity. Last, the overall severity of OCD symptoms is rated using the same three items and a global (0–15) measure of impairment. DY-BOCS has showed good internal consistency and external validity in previous studies using both interview-only and self-report/interview versions [23, 26–28]. Based on the content of the severity questions, a DY-BOCS score equal to or above 5 indicates clinically significant symptoms, which is in line with the best breakpoint for clinical significance on the CY-BOCS [29, 30] from which the DY-BOCS originated. The Swedish and Spanish versions of DY-BOCS used in this study have been evaluated in prior research using samples of youth with OCD from both countries [26, 28], indicating preserved psychometric properties compared to the original version [23].

### Statistical Analysis

Group differences were examined using t-tests, chi-squared tests, and Mann-Whitney U tests. Using the R library *lavaan*, we fitted the proposed six-dimension model to data from Sweden and Spain, respectively. Fit indices were evaluated to assess whether fit was adequate and fit indices for the six-factor model were compared with those of a one-factor model where covariance among items is explained by a single overarching OCD factor. The following fit indices were evaluated: Confirmatory Fit Index (CFI), Root Mean Square Error of Approximation (RMSEA), Standardized Mean Square Residual (SRMR), and Tucker-Lewis fit Index (TLI). Adequate model fit is indicated higher CFI/TLI (values > 0.90 are indicative of adequate fit), and lower RMSEA and SRMR (values < 0.06 and 0.08, respectively, are indicative of good fit) [31]. To examine whether it was justified to pool the samples from Spain and Sweden for breakpoint examination, we tested for measurement invariance which evaluates whether the same psychometric constructs are tested across groups. Measurement invariance was tested within *lavaan*, using a line of nested models with each additional model having more equality constraints across groups. A three-step approach was used. First, we tested for configural invariance. If this model met the fit criteria described above, we tested for metric invariance and used the configural model as the referent comparison model. If metric invariance was established (i.e., a reduction below 0.01 on the CFI index), we tested for strict invariance and again interpreted a reduction below 0.01 on the CFI index as an indicator of invariance. Diagonally weighted least squares estimation was used and robust fit indices were computed in all analyses. We also examined whether associations between the OCI-CV and DY-BOCS dimensions were similar in the two countries by comparing correlation coefficients (Spearman’s rho).

To derive the best OCI-CV breakpoints for clinically significant symptoms within each symptom dimension, receiver-operating characteristic (ROC) analyses were used. To facilitate interpretation of the area under the curve (AUC) values for each OCI-CV subscale, we interpreted AUC values from 0.7 to 0.8 as acceptable, from 0.8 to 0.9 as excellent and above 0.9 as outstanding. The Youden index was used to identify the best breakpoints and because those with versus without clinically significant symptoms within each DY-BOCS dimension were about evenly distributed (see Table 1), we used accuracy as a measure of precision for the derived breakpoints.

## Results

Statistically significant differences between countries for Table 1 variables were present for age (*t*[195] = 3.76, *p* < .001), proportion with clinically significant disturbing thoughts/checking symptoms (X^2^[1] = 6.86, *p* = .01), severity on the OCI-CV doubting/checking scale (U = 5842.0, *p* = .01), severity on the OCI-CV obsessing scale (U = 5648.0, *p* = .04), severity on the OCI-CV washing scale (U = 5785.0, *p* = .02), severity on the OCI-CV ordering scale (U = 5714.5, *p* = .03), and severity on the OCI-CV neutralizing scale (U = 5922.5, *p* = .01). All differences for clinical variables indicated higher severity in the Swedish group.

Model/data fit of the original OCI-CV six-factor structure was adequate in each country (except for the SRMR index) and superior to model/data fit of a one-factor structure, which showed very poor fit. Fit indices for both models using both samples are presented in Table 2. The internal consistency (Cronbach’s alpha) for the items of each subscale of the six-factor structure was adequate for doubting/checking (Sweden = 0.87; Spain = 0.87), obsessing (Sweden = 0.82; Spain = 0.82), washing (Sweden = 0.90; Spain = 0.86), ordering (Sweden = 0.92; Spain = 0.92), and hoarding (Sweden = 0.85; Spain = 0.86) but not for neutralizing (Sweden = 0.68; Spain = 0.74). Strict invariance across countries was found for the six-factor structure (detailed invariance results are presented at the bottom of Table 2).

**Table 2:** Fit indices of the six-factor OCI-CV structure using the Swedish and Spanish samples

	Df; χ2	*p*	CFI	ΔCFI	TLI	RMSEA	SRMR
Six-factor structure							
Sweden	189; 215.9	0.02	0.967	–	0.960	0.049	0.097
Spain	189; 213.4	0.02	0.978	–	0.973	0.049	0.092
One-factor structure							
Sweden	174; 715.1	< 0.001	0.582	–	0.535	0.167	0.212
Spain	174; 603.3	< 0.001	0.764	–	0.738	0.152	0.200
Measurement invariance							
Configural	348; 429.4	0.002	0.973	–	0.968	0.049	0.094
Scalar	363; 432.1	0.007	0.977	+ 0.004	0.974	0.044	0.096
Strict	399; 475.0	0.005	0.975	− 0.002	0.974	0.044	0.096

*OCI-CV* Obsessive-Compulsive Inventory – Child Version

There was a statistically significant correlation between DY-BOCS-assessed disturbing thoughts/checking symptoms and self-reported scores on the OCI-CV scale of obsessing in both countries (Spain: *r* = .47, *p* < .001; Sweden: *r* = .34, *p* < .001). Using the Spanish sample, disturbing thoughts/checking also correlated significantly with washing (*r* = .25, *p* = .01), and using the Swedish sample with doubting/checking (*r* = .37, *p* < .001) and neutralization (*r* = .24, *p* = .02). DY-BOCS assessed symmetry/ordering symptoms correlated significantly with the OCI-CV ordering scale in both countries (Spain: *r* = .40, *p* < .001; Sweden: *r* = .46, *p* < .001). Using the Spanish sample, it also correlated significantly with doubting/checking (*r* = .28, *p* = .01) and neutralization (*r* = .24, *p* = .02), and using the Swedish sample with hoarding (*r* = .31, *p* < .01) and neutralization (*r* = .34, *p* < .001). DY-BOCS assessed contamination/cleaning symptoms correlated significantly with the OCI-CV washing scale in both countries (Spain: *r* = .58, *p* < .001; Sweden: *r* = .71, *p* < .001). Using the Spanish sample, it also correlated significantly with obsessing (*r* = .29, *p* < .01).

**Fig. 1:**
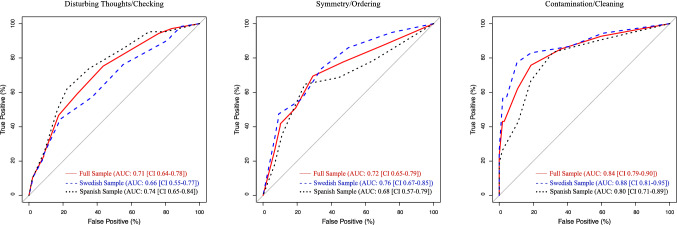
ROC curves for the OCI-CV scale of obsessing in relation to clinically significant disturbing thoughts/checking symptoms (left), ordering in relation to symmetry/ordering (middle), and washing in relation to contamination/cleaning (right)

In Table 3, we present the performance of the best breakpoints for discriminating between those with versus without clinically significant symptoms within the three major symptom dimensions of OCD. For the disturbing thoughts/checking factor, the only OCI-CV scale that reached an acceptable AUC value was the obsessing scale. A score equal to or above 4 points on this scale generated a sensitivity of 75% and a specificity of 65% and the accuracy was 67%. For symmetry/ordering, the only OCI-CV scale that reached an acceptable AUC value was the ordering scale. A score equal to or above 3 points on this scale generated a sensitivity of 69% and a specificity of 71% and the accuracy was 70%. For contamination/cleaning, the OCI-CV washing subscale showed an excellent AUC value. A score equal to or above 3 points on this scale generated a sensitivity of 76% and a specificity of 82% and the accuracy was 79%. The ROC curves for the OCI-CV scales that reached acceptable AUC values are shown in Fig. 1.

**Table 3:** Breakpoints, sensitivity and specificity and AUC for the OCI-CV dimensions in relation to clinician-rated severity within the OCD dimensions of forbidden thoughts/checking, taboo thoughts, symmetry/ordering, and contamination/cleaning. AUC values above 0.70 are highlighted in bold

	Classification based on clinician-rated symptom dimension severity								
	Disturbing thoughts/checking			Symmetry/Ordering			Contamination/Cleaning		
	AUC (95% CI)	Breakpoint value >=	Sensitivity/ specificity (%)	AUC (95% CI)	Breakpoint value >=	Sensitivity/ specificity (%)	AUC (95% CI)	Breakpoint value >=	Sensitivity/ specificity (%)
OCI-CV dimensions									
Doubting/Checking	0.65 (0.58–0.73)	4	71/52	0.58 (0.50–0.66)	7	31/88	0.58 (0.50–0.66)	5	55/59
Obsessing	**0.71** (0.64–0.78)	4	75/56	0.51 (0.43–0.59)	2	88/17	0.59 (0.51–0.67)	7	24/91
Washing	0.56 (0.48–0.65)	1	82/32	0.56 (0.48–0.64)	0	0/100	**0.85** (0.80–0.90)	3	76/82
Hoarding	0.55 (0.48–0.63)	2	55/53	0.61 (0.53–0.69)	2	61/61	0.52 (0.44–0.60)	4	16/85
Ordering	0.50 (0.42–0.58)	3	53/51	**0.72** (0.65–0.79)	3	69/71	0.50 (0.42–0.58)	1	83/25
Neutralizing	0.60 (0.53–0.68)	1	73/43	0.63 (0.50–0.71)	2	49/72	0.50 (0.43–0.58)	1	69/38

*AUC* Area under the Curve, *OCD* Obsessive-Compulsive Disorder, *OCI-CV* Obsessive-Compulsive Inventory – Child Version

To see whether the proposed breakpoints worked adequately in each country, we examined the accuracy of the breakpoints in the two countries separately. A breakpoint of 4 on the OCI-CV obsessing scale was 65% accurate in classifying individuals with clinically significant disturbing thoughts/checking symptoms in the Swedish sample and 69% accurate in the Spanish sample. A breakpoint of 3 on the OCI-CV ordering scale was 70% accurate in classifying individuals with clinically significant symmetry/ordering symptoms in the Swedish sample and 70% accurate in the Spanish sample. A breakpoint of 3 on the OCI-CV washing scale was 82% accurate in classifying individuals with clinically significant contamination/cleaning symptoms in the Swedish sample and 76% accurate in the Spanish sample.

Last, we examined how a more conservative breakpoint affected the positive predictive value (PPV; i.e., the proportion of those identified as cases that were true cases). When using a breakpoint of ≥ 4 on the washing scale, the sensitivity was 63% and the specificity was 90%, making the PPV 86%, showing that 86% of all participants above the breakpoint were true cases. This should be compared to a PPV of 80% for the 3-point breakpoint. Raising the breakpoint for the obsessing subscale to ≥ 5 generated a sensitivity of 59% and a specificity of 72%, making the PPV 71% (compared to a PPV of 66% for the 4-point breakpoint). Raising the breakpoint for the ordering subscale to ≥ 4 generated a sensitivity of 51% and a specificity of 81%, making the PPV 77% (compared to a PPV of 74% for the 3-point breakpoint).

## Discussion

We examined whether self-reported scores on OCI-CV from youth with OCD can be used to adequately capture clinical severity within each of the three major symptom dimensions of OCD. Our findings suggest that the OCI-CV washing scale has good precision to identify those with clinically significant contamination/cleaning symptoms. Recent work has shown that contamination/cleaning symptoms in youth with OCD are motivated by disgust rather than fear [7, 8], and contamination/cleaning symptoms in adults are linked to better neuropsychological performance on planning and response inhibition tasks [32]. Further, behavioral genetic work indicates that partly different genetic etiologies underlie the major symptoms of OCD [13]. Thus, the literature suggests that contamination-related OCD may be underpinned by mechanisms that are partially specific for this type of OCD. The identification of such mechanisms is crucial to prevent and treat the disorder effectively. Currently, there is no well-established method to classify youth with OCD with clinically significant contamination/cleaning symptoms and to the best of our knowledge, the DY-BOCS interview is the only available dimensional measure for youth with OCD that respects the factor analysis-based symptom structure of the disorder. In the absence of DY-BOCS, which is time consuming and only available in a small number of languages, OCI-CV can now be considered a viable option. A multitude of research questions can be addressed using the OCI-CV breakpoint provided in this study, including questions about whether those with primary contamination/cleaning symptoms respond differently to treatment.

The ordering scale of the OCI-CV showed adequate precision to identify those with clinically significant symmetry/ordering symptoms. The symmetry/ordering dimension of pediatric OCD is poorly understood and research suggests that the motivation behind symmetry symptoms are not to reduce anxiety but instead to relieve a sense of incompleteness or not-just-right feelings [7, 8]. Up until now there has been no consensus on how to best classify youth with clinically significant symmetry symptoms and different versions of the most common symptom measure – the symptom checklist of the C/Y-BOCS [33] – contains none or only a few yes/no symmetry items, which further complicates adequate classification. The present study provides guidance in this respect and OCI-CV can now be used to obtain a reasonable valid classification. Our results also showed that OCI-CV can be used to adequately identify those with significant disturbing thoughts/checking symptoms. In line with the ordering scale, the accuracy of this breakpoint was somewhat lower than what was found for the washing scale, but still in the acceptable range.

It is possible that researchers under some circumstances want to create purer groups of clinical cases. We showed that by adding one point to the established breakpoints (i.e., using 4 points on the OCI-CV washing scale, 5 on the obsessing scale, and 4 on the ordering scale), the PPV increased slightly for all major dimensions, with 86% in the contamination/cleaning group being true cases, and 71% and 77% in the disturbing thoughts/checking and symmetry/ordering groups, respectively.

The hoarding subscale of OCI-CV could not be used to classify symptom severity within any major OCD dimension which is not surprising given that hoarding is now considered a separate disorder [5]. The OCI-CV neutralizing scale showed similar results. The items of the neutralizing scale have showed low internal consistency in previous research [16, 20] and did so also in this study. The present study adds to the literature by showing that the neutralizing scale may be of limited use also when identifying the major symptom dimensions of pediatric OCD. However, in the individual case, high scores on the neutralizing OCI-CV items may be clinically important and inform treatment. Another OCI-CV subscale that could not be used to adequately identify clinically significant symptoms within any major symptom dimension was the doubting/checking scale. Doubt has been described as a core element of OCD [34] and empirical results support this assumption [35]. However, doubting and checking may be involved in symptoms across different major symptom dimensions (e.g., hand washing, ordering rituals, checking behaviors) which may explain why it was not adequate to identify patients within a specific dimension. A closer look at our results supports this as doubting/checking indeed was positively related to all three symptom dimensions (AUC was above 0.50 and the confidence interval did not include zero) but its precision was low.

This study was limited by the use of data from only two countries, and it is warranted to examine whether similar breakpoints apply in other countries and contexts. Another limitation is the inherent limitations of the reference measure, the DY-BOCS. While DY-BOCS benefits from being interview-led (which increases the chance to thoroughly explain symptom content), it is not a perfect measure of OCD dimensionality as it still suffers from possible under- and over-reporting. Under-/over-reporting are difficulties attached to all interview/self-report measures and cannot be overcome until purely objective measures of symptom dimensions exist. It is unclear whether objective psychiatric measures will or even can (on conceptual grounds) be developed [36], but future research may want to explore if other ways to measure symptoms can aid to more precise measurement of OCD symptoms. A promising method is provided within the framework of ecological momentary assessment [37]. Last, the breakpoints presented in the present study cannot be extrapolated to broader clinical groups as only patients with a lifetime history of OCD were included, of which a vast majority had ongoing OCD. Previous work with broader clinical groups has shown that the OCI-CV ordering scale does not contribute uniquely to OCD status [21] and empirical evidence points towards that the items of OCI-CV may be interpreted differently by clinical and non-clinical participants, with lower internal consistency of the subscales in non-clinical samples [35].

## Summary

Self-reported scores on the OCI-CV from youth with pediatric OCD can be used to adequately capture those with clinically significant symptoms in each of the three major symptom dimensions of OCD. OCI-CV works best for contamination/cleaning symptoms but can be used with moderate confidence also regarding disturbing thoughts/checking and symmetry/ordering symptoms. Possible areas where these benchmarks can be used are in the investigation of mechanisms involved in pediatric OCD and in predicting long- and short-term outcome.
